# Generalization and False Memory in an Acquired Equivalence Paradigm: The Influence of Physical Resemblance Across Related Episodes

**DOI:** 10.3389/fpsyg.2021.669481

**Published:** 2021-08-20

**Authors:** Caitlin R. Bowman, Maria-Alejandra de Araujo Sanchez, William Hou, Sarina Rubin, Dagmar Zeithamova

**Affiliations:** ^1^Department of Psychology, University of Oregon, Eugene, OR, United States; ^2^Department of Psychology, University of Wisconsin-Milwaukee, Milwaukee, WI, United States

**Keywords:** acquired equivalence associative learning task, generalization, inference, associative memory, source memory, false memory

## Abstract

The ability to make inferences about related experiences is an important function of memory that allows individuals to build generalizable knowledge. In some cases, however, making inferences may lead to false memories when individuals misremember inferred information as having been observed. One factor that is known to increase the prevalence of false memories is the physical resemblance between new and old information. The extent to which physical resemblance has parallel effects on generalization and memory for the source of inferred associations is not known. To investigate the parallels between memory generalization and false memories, we conducted three experiments using an acquired equivalence paradigm and manipulated physical resemblance between items that made up related experiences. The three experiments showed increased generalization for higher levels of resemblance. Recognition and source memory judgments revealed that high rates of generalization were not always accompanied by high rates of false memories. Thus, physical resemblance across episodes may promote generalization with or without a trade-off in terms of impeding memory specificity.

## Introduction

Memory integration—the ability to link information across related experiences—is an important function of memory. It allows individuals to build knowledge to support inferences and generalize prior experience to novel situations. For example, after hearing that your friend from New York, Kyle, is spending his summer vacation at the Jersey Shore, you may generalize this preference to another friend from New York, Brad, assuming that he might make similar vacation plans. Memory generalization, however, may come at the expense of memory specificity, as integration may lead to false memories (Carpenter and Schacter, 2017, 2018). That is, you might falsely remember that both Kyle and Brad told you that they were going to the Jersey Shore when, in fact, you had merely inferred Brad's plans. In this study, we were interested in the relationship between false memories and generalization, and whether contexts that promote false memories also tend to promote generalization in decision-making.

**Figure 1:**
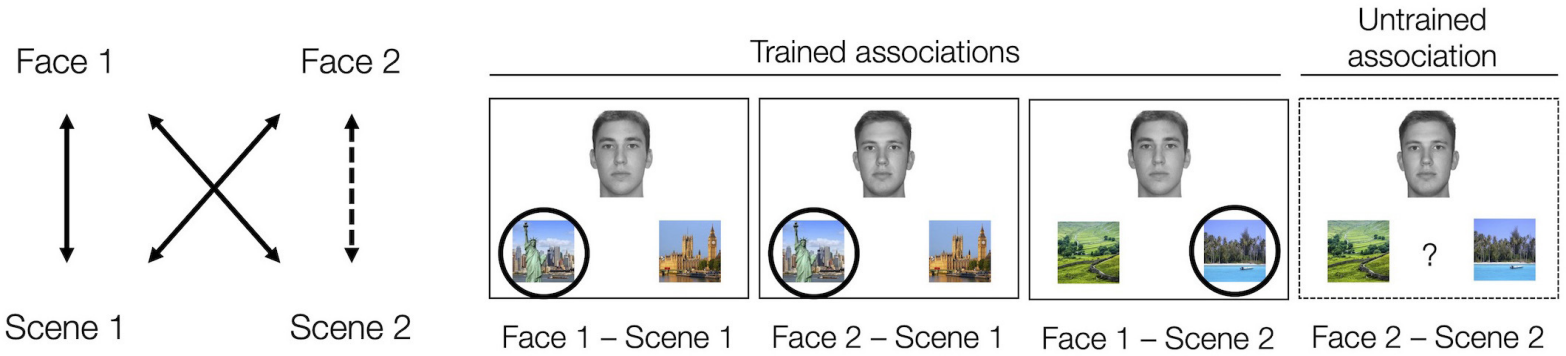
Acquired equivalence task structure. Participants learn that two people (Face 1 and Face 2) share one association (in our task, living in the same city, e.g., New York not London) via feedback-based learning. They also learn an additional association for Face 1 (in our task, their preferred vacation location, e.g., the beach, not the countryside) also via feedback. The correct scene association for each face is indicated by a circle (Scene 1 and Scene 2). The other scene is a foil scene (Scene 1' and Scene 2'). After training, participants are tested on these trained associations as well as on the untrained Face 2 – Scene 2 association (dashed line, dashed border). Participants were not informed of the structure of the task set nor that they would be tested on associations not present in the training phase. The tendency to say that Face 2 is associated with Scene 2 over Scene 2' (e.g., the beach over the countryside) is taken as evidence of acquired equivalence between Face 1 and Face 2. All the faces depicted come from the Dallas Face Database (Minear and Park, 2004).

Acquired equivalence is one form of generalization, which involves assuming that when a pair of stimuli share one commonality (e.g., two people who are both from New York), they are likely to share other characteristics (e.g., preferred vacation location) (Edwards et al., 1982; Honey and Hall, 1989; Shohamy and Wagner, 2008). Importantly, acquired equivalence occurs when characteristics are extended from one individual to the other through inference rather than through direct learning. In this paradigm (see Figure 1), participants generally undergo training to learn a set of associations, like that Face 1 and Face 2 both prefer Scene 1 over Scene 1′. They also learn that Face 1 prefers Scene 2 over Scene 2′. The sets of associations that the participants learn directly through training are known as the trained associations. The participants are subsequently tested on the trained associations as well as on the critical untrained association between Face 2 and Scene 2. When the participants indicate that Face 2 is associated with Scene 2 over Scene 2′ at rates reliably above 50%, it is taken as evidence that the participants have generalized across Face 1 and Face 2, showing “acquired equivalence” between them.

Integrative encoding is one mechanism by which acquired equivalence can occur. It involves combining memory representations of events at the time of learning based on their commonalities (Shohamy and Wagner, 2008; Zeithamova et al., 2012a; Richter et al., 2016; Schapiro et al., 2017). For example, meeting Brad and hearing he is from New York may trigger a memory for Kyle because of their shared association with New York. The memory representations of Brad and Kyle may then become integrated, because they were active at the same time, making it more likely that new information learned about either person will be applied to both of them. Prior study has supported the notion that acquired equivalence can be a function of integrative encoding, showing that acquired equivalence judgments can be made as quickly as judgments about associations learned directly (Shohamy and Wagner, 2008; but see de Araujo Sanchez and Zeithamova, 2020). Inferences do not always require additional processing time at retrieval suggests that the inferred relationship can be established prior to retrieval. Further, hippocampal processing at the time of encoding predicted later generalization, suggesting that links across episodes were formed during learning rather than on-the-fly during generalization itself (Shohamy and Wagner, 2008).

A key component of integrative encoding is pattern completion. Pattern completion occurs when a partial cue triggers retrieval of a complete engram, leading to recognition (Marr, 1971; McClelland et al., 1995; Hunsaker and Kesner, 2013; Horner et al., 2015). In the above example, pattern completion occurs when Brad mentions being from New York, which then triggers a memory for Kyle who is also associated with New York. This process can lead to integrative encoding when reactivated prior events are then re-encoded along with current experience (Zeithamova et al., 2012a; Schapiro et al., 2017). Further, a prior study suggests that the likelihood of pattern completion occurring increases with the degree of resemblance between past experience and the current retrieval cue (Guzowski et al., 2004; Lee et al., 2004; Vazdarjanova and Guzowski, 2004; Liu et al., 2016). Recent evidence has also shown that higher similarity between related episodes increases the likelihood that individuals will make new inferences that combine information across those episodes (Molitor et al., 2021). The first goal of this study was to extend this finding to an acquired equivalence paradigm, testing whether increasing physical resemblance between stimuli comprising overlapping associations would lead to increases in rates of acquired equivalence.

While integrative encoding may support inference and facilitate later generalization, it may also come with a trade-off: reduced memory specificity. Combining representations of related events may lead unique aspects of those experiences to be lost or conflated. Increases in false memories, in particular, have sometimes been posited as a consequence of generalization (Carpenter and Schacter, 2017, 2018; Varga et al., 2019). For example, Carpenter and Schacter (2017, 2018) showed that when participants made inferences across related experiences, they tended to misattribute unique contextual details from one episode to the other, conflating the two experiences. Likewise, Shohamy and Wagner (2008) reported anecdotal evidence that individuals who generalized well in an acquired equivalence paradigm were those who conflated directly learned and inferred relationships during learning, although this finding has been difficult to replicate (de Araujo Sanchez and Zeithamova, 2020). Outside inference paradigms, prior research shows that rates of false memories tend to increase with increasing perceptual and/or conceptual overlap between new and old information (Roediger et al., 1995; Arndt and Reder, 2003; Gutchess and Schacter, 2012; Bowman and Dennis, 2015). This effect is thought to occur, in part, because pattern completion can be triggered by the partial overlap between new and old information (Toner et al., 2009; Yassa et al., 2011; Vieweg et al., 2015). Thus, while manipulating the physical resemblance across stimuli within an acquired equivalence paradigm may lead to more generalization by promoting integrative encoding, it may also lead to higher rates of false memories.

Alternatively, generalization may not lead to false memories if inferences are retrieval-based rather than formed through integrative encoding (Zeithamova and Bowman, 2020). Retrieval-based inference occurs when related episodes are encoded separately and then retrieved in parallel to make generalization decisions on the fly (Zeithamova and Preston, 2010; Banino et al., 2016). Importantly, when generalization occurs *via* this flexible retrieval mechanism, it is possible to maintain separate representations of related episodes while still generalizing. In this case, memory specificity and generalization may go hand-in-hand, because the quality of the separate representations determines the accuracy of later decision-making (Kumaran, 2012; Kumaran and McClelland, 2012). Recent study has formally tested both generalization and memory specificity in inference paradigms, sometimes showing no trade-off (de Araujo Sanchez and Zeithamova, 2020), or even a positive relationship between them (Banino et al., 2016). Thus, false memories are not always a consequence of generalizing, and the degree of overlap across experiences may have different effects on false memory and generalization when related experiences are coded separately (Zeithamova and Bowman, 2020).

To investigate the parallels between memory generalization and false memories, we conducted three experiments using an acquired equivalence paradigm while manipulating the physical resemblance between items that comprised related experiences using computer-blended face stimuli. In Experiment 1, some pairs of faces shared a common parent image, while other pairs were blended without a shared parent. In Experiment 2, all pairs of faces shared a common parent, but the weight given to the shared parent varied parametrically. We then tested both generalization and rates of false memory for the source of the inferred information, measuring whether physical resemblance had similar or distinct effects across these two memory judgments. In Experiment 3, we added a recognition test to determine whether pair-mate faces could be discriminated from one another. We hypothesized that physical resemblance would increase the likelihood that prior related experiences would be reactivated during new learning, promoting integrative encoding and supporting generalization. However, we also hypothesized that reliance on integrative encoding would lead to conflating of separate experiences, with higher rates of false memories for the source of information when items resembled one another.

## Experiment 1

### Materials and Methods

#### Participants

Forty participants from the University of Oregon completed the experiment for course credit (27 females, mean age = 19.5 years, SD age = 1.3 years, age range = 18–23 years). This sample size was within the range of prior acquired equivalence studies collected in the laboratory (de Araujo Sanchez and Zeithamova, 2020). While there was no prior study on which to base the expected effect size of physical similarity, we chose a moderate effects size (d = 0.5) *a priori* as the effect size for which we aimed to have sufficient power. All the participants completed written informed consent, and the Institutional Review Board of the University of Oregon approved all procedures.

#### Materials

The stimuli were eight colored images of scenes and eight gray scale images of Caucasian male faces. Faces and scenes were paired to form four quadruplets. Each quadruplet included two faces (Face 1 and Face 2, called pair-mates) each paired with two scenes (Scene 1 and Scene 2). There were four possible face-scene pairs within each quadruplet: Face 1-Scene 1, Face 1-Scene 2, Face 2-Scene 1, and Face 2-Scene 2. Three of these face-scene pairs were used for training (Face 1-Scene 1, Face 1-Scene 2, and Face 2-Scene 1). The last one (Face 2-Scene 2) was untrained and only used during the subsequent test to measure generalization *via* acquired equivalence (Figure 1). Of the eight colored scenes, four were well-known cities and four were nature scenes. For each quadruplet, Scene 1 was a city scene and Scene 2 was a nature scene. The specific pairing of scenes to quadruplets was randomly assigned for each participant.

**Figure 2:**
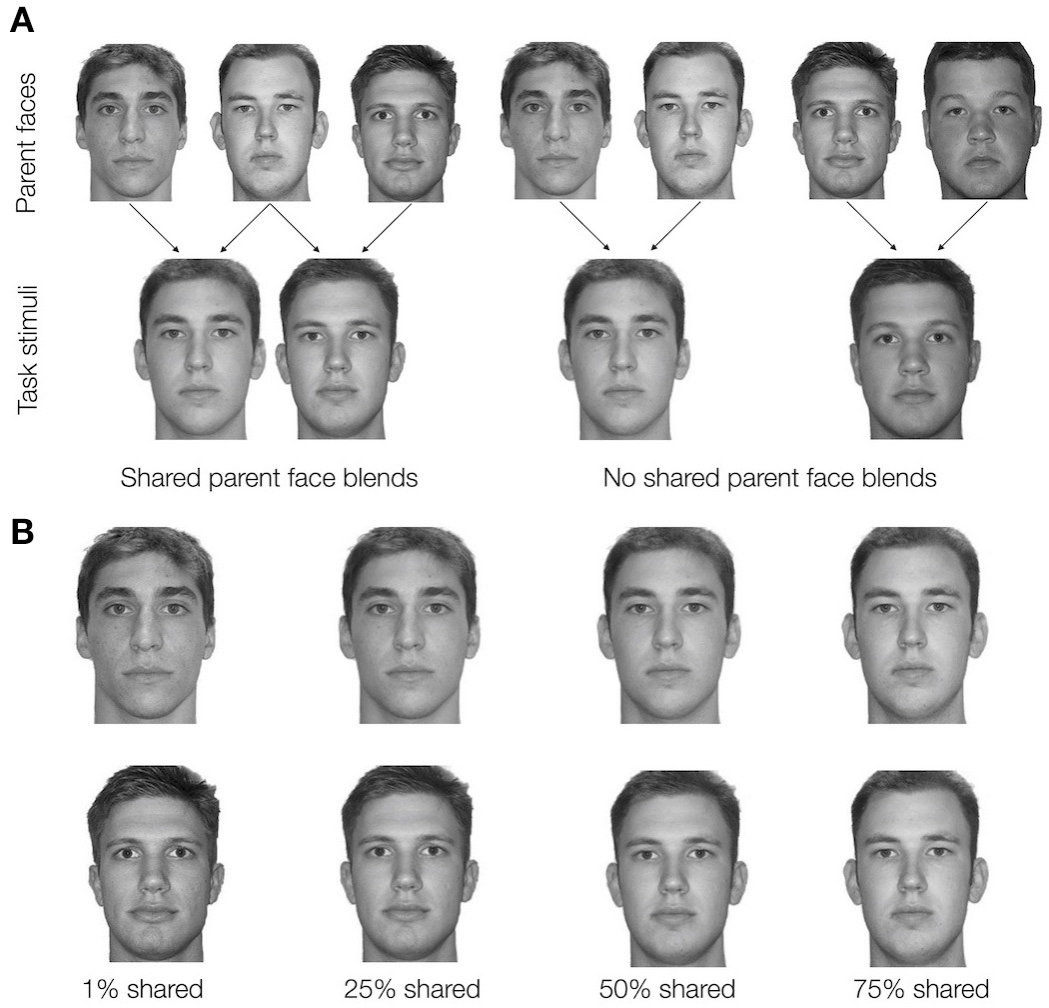
**(A)** In Experiment 1, pairmate faces were blended to either share a parent face or have no shared parent face. Example unaltered parent faces (not shown during any phase of the experiment) are depicted in the top row. On the left, the faces are combined to make shared parent pairmates. On the right, a similar set of unaltered faces are blended to make no shared parent pairmates. Arrows indicate which parent faces would be blended to create task stimuli. All task stimuli in Experiment 1 were 50/50 blends of their parent faces. **(B)** In Experiment 2, all pairmate faces were blended with a shared parent face but the percentage of the shared parent face included in the final blend face varied parametrically (1%, 25%, 50%, or 75% shared parent). Here, an example Face 1 – Face 2 pair (the shared parent example from **A)** is shown at each of the four possible blend levels. The top row depicts an example Face 1 at each blend level. The bottom row depicts an example Face 2 at each blend level. In **A** and **B**, the same faces are shown for different levels of similarity to demonstrate how the blending process manipulates similarity. In the experiment, a given unaltered face would be part of only one Face 1 – Face 2 pairmate. All the faces depicted come from the Dallas Face Database (Minear and Park, 2004).

Faces within each quadruplet were constructed by blending two unaltered face images together using FantaMorph Version 5 by Abrosoft. For each participant, the pair-mate faces for two of the four quadruplets were blended with a shared parent that made up 50% of the blend (Figure 2A). For example, unaltered Face A would be 50/50 blended with unaltered Face B to create Face 1, and unaltered Face C would also be 50/50 blended with unaltered Face B to create Face 2. Thus, these pair-mate faces were manipulated to share a physical similarity. The faces in the other two quadruplets were also created as 50/50 blends but did not share a parent face. For example, unaltered Face D would be 50/50 blended with unaltered Face E to create Face 1, and unaltered Face F would be 50/50 blended with unaltered Face G to create the Face 2. These pair-mates will be referred to as no shared parent pair-mates. For each participant, 14 unaltered faces were randomly selected from a set of 18 possible faces and randomly assigned to create shared parent pair-mates (six parent faces, three for each of two shared parent pair-mates) or no shared parent pair-mates (eight parent faces, four for each of two no shared parent pair-mates). The resulting blended faces were then randomly paired with their nature/city scenes.

#### Procedure

##### Initial Exposure

To familiarize the participants with the faces prior to asking them to form associations between faces and scenes, the participants first passively viewed each face in isolation three times across a single block. Each face was presented for 2 s followed by a 1-s fixation cross. The participants made no responses and were simply instructed to remember the faces without any indication of the upcoming task structure.

##### Training

During training, the participants learned three face-scene associations for each quadruplet: Face 1-Scene 1, Face 2-Scene 1, and Face 1-Scene 2. They were instructed to try to learn where each person vacationed (nature scene) and where they lived (city scene). On each trial, a face cue was presented at the top of the screen with two scene options below. For nature scene trials, the cue “Where does he vacation?” appeared at the top of the screen above the face. “Where does he live?” appeared on the screen for city scene trials. The face and scene options were presented for 3 s during which time the participants selected which of the two presented scenes they thought was associated with the cue face by pressing a button on the keyboard. The participants were then given feedback on a separate screen: “Correct” if they selected the correct scene, “Incorrect” if they selected the incorrect scene, and “Too late” if they did not respond within 3 s. Feedback appeared on the screen for 1 s, followed by a 1-s fixation cross.

The scene options were generated so that (1) options were either both city scenes or both nature scenes and (2) scenes were paired together so that a given scene was always presented as an option along with the same other scene, once serving as the target (correct) scene and once as the foil (incorrect) scene. For example, for quadruplet A, the nature scene options might be a beach and a field with the beach being the correct answer. The city scene options might be New York and London with New York being the correct answer. Quadruplet B would then be yoked to Quadruplet A such that it has the same scene options for both nature and city scenes, but the opposite correct answers (i.e., the field and London). This ensures that the participants must learn the association between the face and the scene and not merely which scene is correct more often or correct when compared with a particular other scene, which could occur if the foil scene was randomized. The left/right presentation of the scene options was counterbalanced within the participants across trials.

The participants underwent 16 blocks of training containing 12 trials each, totaling 192 training trials. They took a self-paced break between each block. The order of trials was randomized for each participant with no cue face-scene option combination shown more than three times consecutively.

##### Acquired Equivalence Test

Immediately following training, the participants were tested on the trained pairs as well as the untrained Face 2-Scene 2 pairing to test for acquired equivalence between Face 1 and Face 2. As in the training phase, a face cue was presented with two scene options on each trial with a corresponding question (“Where does he live?” or “Where does he go on vacation?”) presented above the cue face. The cue face and scene options were presented for 3 s during which time the participants were to indicate which scene was associated with the cue face. Unlike the training phase, the participants did not receive feedback during the test. Each trial ended with a fixation cross that was presented for 1 s. The structure of the scene options remained the same as in the training phase.

To improve the reliability of estimates of acquired equivalence given the limited trials (one for each of four quadruplets), each association was tested six times throughout the test (Shohamy and Wagner, 2008). The order of presentation was randomized with the constraint that the same association was not tested more than twice in a row. The acquired equivalence test was completed in a single block with a total of 96 trials (four associations for each of four quadruplets each presented six times).

##### Source Memory Test

Following the acquired equivalence test, the participants underwent a source memory test to measure how well they were able to remember when they encountered each association. On each trial, the participants were presented with one face and two scenes arranged vertically above each other to remove the left-right organization of scenes present in training and test. The task was to indicate whether and when the three pictures were presented together previously, regardless of their spatial arrangement.

There were four possible response options, presented from left to right: “study,” “test,” “both,” and “never.” “Study” responses indicated that the images were seen together only during the training phase—an answer that was never correct but was included so that the response options would not give away the task structure. “Test” responses indicated that the images were seen together only during the acquired equivalence test the participants had just completed—an answer that was correct only for Face 2-Scene 2 acquired equivalence items. “Both” responses indicated that the images had been seen together during both the training phase and the acquired equivalence test—an answer that was correct for the three types of trained pairs (i.e., Face 1-Scene 1, Face 2-Scene 1, and Face 1-Scene 2). Lastly, “never” responses indicated that the three images had not been shown together in previous phases of the experiment. We constructed three types of recombined trials for which “never” was the correct answer. “Recombined all” trials consisted of a face and two scenes in which none of the components had been presented with one another. “Recombined face” trials consisted of two scenes that had been previously presented together (e.g., Scene 1 and Scene 1′) and a face that was never presented with those scenes. “Recombined scene” trials consisted of a cue face presented with its corresponding Scene 1 and Scene 2. Thus, each scene was presented with the face previously, but the scenes were never presented together. While this trial type has been used in a prior study with random scene types (de Araujo Sanchez and Zeithamova, 2020), it was particularly obvious in this experiment that these images had not been presented together because of the nature vs. city scene manipulation, as scene options were always either two cities or two nature scenes. One face from each of the four quadruplets was used to create all seven types of source memory trials: three trained (“both”), 1 untrained (“test”), and three recombined (“never”) for a total of 28 source memory test trials. The source memory test was self-paced, with a 1-s fixation cross between each trial.

Of primary interest in the source memory test was how often the participants reported having seen the untrained association during both training and test. We refer to this as a source false memory, but it could also reflect a participant generating the F2-S2 association during encoding through reactivation, then later misattributing that internal experience to the training task. Other trial types were included to help differentiate integration-related source confusion (i.e., false memory for untrained associations being presented during the study) from overall poor source memory or response biases.

#### Design and Statistical Analyses

The primary independent variable of interest was a physical resemblance between pair-mate faces. There were two levels of physical resemblance (sharing a parent, not sharing a parent), and this was manipulated within subjects. We had two primary dependent variables of interest: acquired equivalence for untrained pairs measured during the acquired equivalence test and false memories for the source of untrained associations. For all analyses, a Greenhouse–Geisser correction was applied (denoted with “GG”) when the sphericity assumption was violated. A Bonferroni correction for multiple comparisons was applied when multiple independent statistical tests were computed, such as following up on a significant omnibus ANOVA with multiple pairwise comparisons.

### Results

#### Training

**Figure 3:**
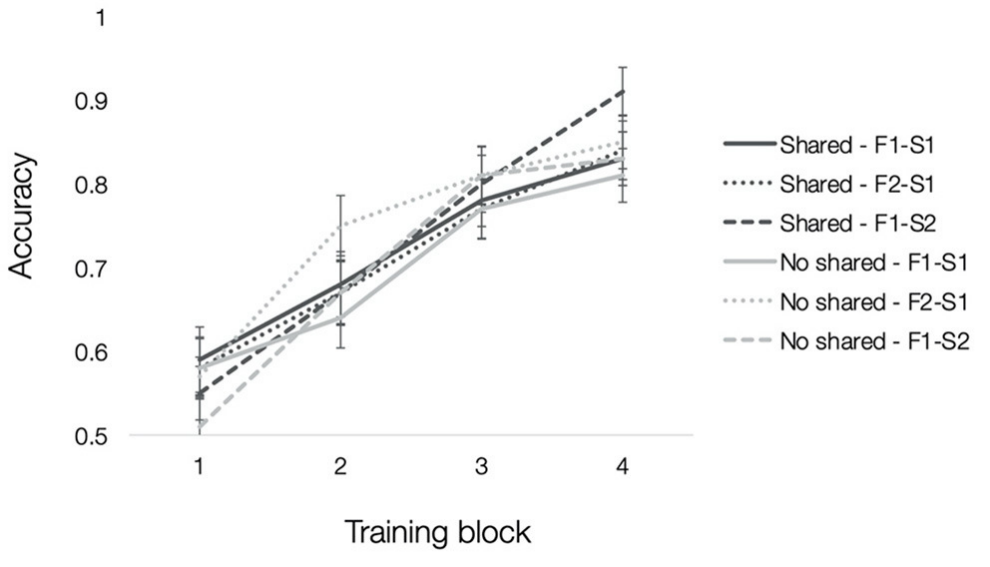
Experiment 1 training accuracy. Mean accuracy from each block of training separated by trained association type (F1-S1 solid lines, F2-S1 dotted lines, F1-S2 dashed lines) and pair-mate type (shared parent in dark gray, no shared parent in light gray). Error bars depict the standard error of the mean across subjects.

Mean accuracies for each type of trained association are depicted in Figure 3. To test how resemblance between faces within a quadruplet affected acquisition of trained associations, we computed a 2 (pair-mate type: shared parent, no shared parent) × 3 (trained association type: F1-S1, F2-S1, and F1-S2) × 4 (training block: 1–4) repeated-measures ANOVA. There was a significant main effect of training block [*F*_(3, 117)_ = 76.89, *p* < 0.001, $\eta_{p}^{2}$ = 0.66] with an accompanying linear effect of training block [*F*_(1, 39)_ = 164.22, *p* < 0.001, $\eta_{p}^{2}$ = 0.81], which showed increasing accuracy with training. No other effects reached significance (all F′s <2, p′s > 0.07).

#### Acquired Equivalence Test

**Figure 4:**
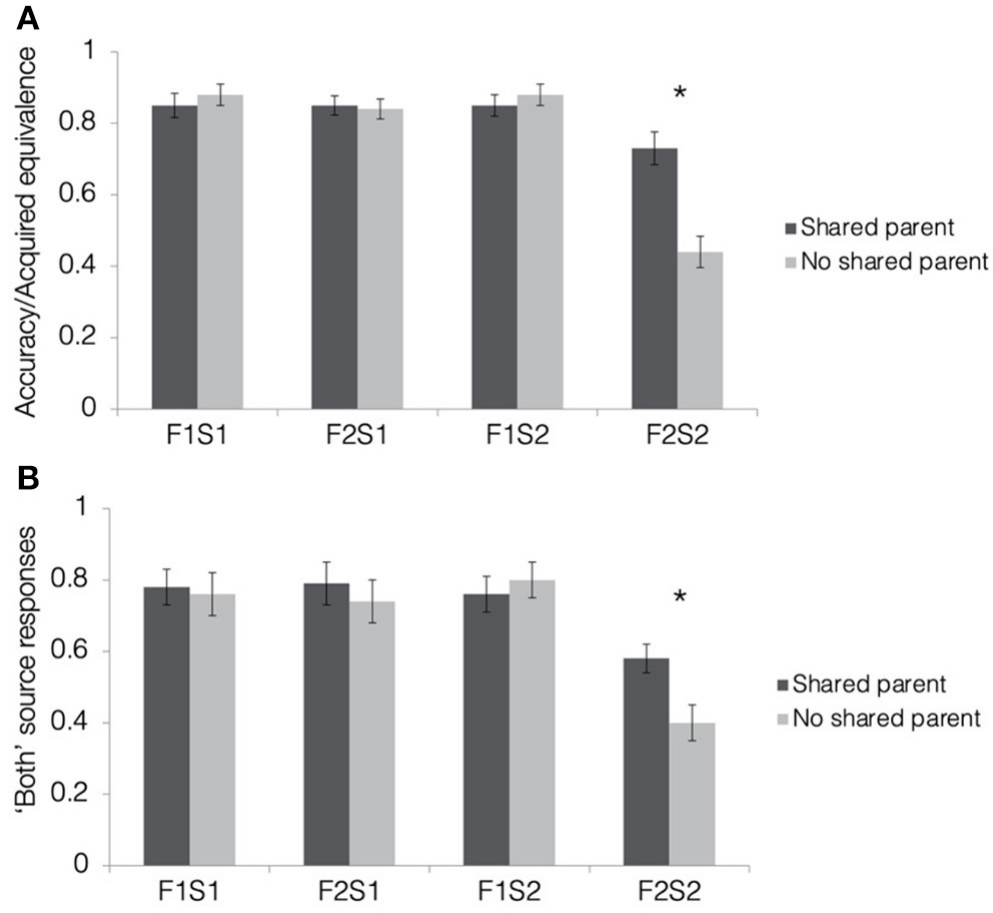
Results from the tests of acquired equivalence and source memory in Experiment 1. **(A)** Acquired equivalence test accuracy for trained associations (F1-S1, F2-S1, and F1-S2) and rates of acquired equivalence for untrained associations (F2-S2). **(B)** For the source memory test, the proportion of trials where the participants responded that they had seen the items together during both study and test. This was the correct response for the trained associations but constituted a false memory for the untrained associations, since they were presented only during the test. All results are depicted separately for quadruplets with faces sharing a parent (dark gray bars) and quadruplets with faces not sharing a parent (light gray bars). Stars indicate a significant paired difference, following a significant test item type × quadruplet type interaction effect (corrected alpha = 0.0125).

Figure 4A depicts results from the acquired equivalence test in terms of accuracy for trained associations (F1-S1, F2-S1, and F1-S2) and the proportion of trials showing acquired equivalence for untrained associations (F2-S2). For the ease of report of the ANOVA results, we will refer to the proportion of untrained associations on which a participant responded consistently with acquired equivalence as “accuracy,” but generalization of scene associations from one face to another is not inherently correct or incorrect in this paradigm. First, we tested whether rates of acquired equivalence differed significantly from chance (50% for two alternative forced choices) using one-sample *t*-tests for shared and no shared parent pair-mates separately. Rates of acquired equivalence were significantly higher than chance for pair-mates sharing a parent [M = 0.73, SD = 0.29; *t*_(39)_ = 4.93, *p* < 0.001, *d* = 0.76; Bonferroni-corrected alpha-level: *p* < 0.025] but not for pair-mates without a shared parent [M = 0.44, SD = 0.28; *t*_(39)_ = −1.46, *p* = 0.15, *d* = −0.23].

To test how resemblance between pair-mate faces affected the tendency to show acquired equivalence, we computed a 2 (pair-mate type: shared parent, no shared parent) × 4 (test item type: F1-S1, F2-S1, F1-S2, and F2-S2) repeated-measures ANOVA. There was a significant main effect of test item type [*F*_(1.94, 75.67)_ = 55.52, *p* < 0.001, $\eta_{p}^{2}$ = 0.59] with all trained associations having higher accuracy (F1-S1: M = 0.86, SD = 0.17; F2-S1: 0.84, SD = 0.13; F1-S2: 0.86, SD = 0.16) than untrained associations (F2-S2: M = 0.58, SD = 0.19; t′s > 8, p′s < 0.001; Bonferroni-corrected alpha level: *p* < 0.0083). Accuracy for the trained associations did not differ from one another (t′s <1, p′s > 0.3). There was also a significant main effect of pair-mate type [*F*_(1, 39)_ = 5.32, *p* = 0.03, $\eta_{p}^{2}$ = 0.12] with higher overall accuracy for shared parent pair-mates (M = 0.82, SD = 0.16) compared with those with no shared parent (M = 0.76, SD = 0.15). These main effects were qualified by a significant pair-mate type × test item type interaction [*F*_(1.73, 67.57)_ = 14.13, *p* < 0.001, $\eta_{p}^{2}$ = 0.27]. There was no difference between shared parent pair-mates and no shared parent pair-mates for any of the trained associations (t′s <1.1, *p* > 0.3), but participants showed higher rates of acquired equivalence when pair-mates shared a parent compared with when they did not [*t*_(39)_ = 4.38, *p* < 0.001, d = 0.69; Bonferroni-corrected alpha level: *p* < 0.0125]. Thus, the participants learned all trained associations equally well, but were more likely to generalize across pairs of faces when they were more physically similar to one another.

#### Source Memory Test

Mean response rates for all source memory response options are presented in Table 1. Mean proportion of “both study and test” responses for each pair-mate and association type is presented in Figure 4B. To test whether the participants tended to falsely remember untrained pairs as having been presented during both the study and test phase, we computed a 2 (pair-mate type: shared parent, no shared parent) × 4 (test item type: F1-S1, F2-S1, F1-S2, F2-S2) repeated-measures ANOVA on the proportion of “both study and test” responses. There was a marginal main effect of pair-mate type [*F*_(1, 39)_ = 3.93, *p* = 0.055, $\eta_{p}^{2}$ = 0.09] with numerically higher rates of “both study and test” responses for pair-mates sharing a parent (M = 0.73, SD = 0.27) compared with those not sharing a parent (M = 0.68, SD = 0.28). There was a significant main effect of test item type [*F*_(1.93, 75.27)_ = 15.25, *p* < 0.001, $\eta_{p}^{2}$ = 0.27, GG] with lower rates of “both study and test” responses for untrained associations (F2-S2: M = 0.49, SD = 0.33) than for all of the trained association types (F1-S1: M = 0.77, SD = 0.34; F2-S1: M = 0.76, SD = 0.35; F1-S2: M = 0.78, SD = 0.3; all t′s > 4, p′s < 0.001). There was no difference between shared parent pair-mates and no shared parent pair-mates for any of the trained associations (t′s <1.1, *p* > 0.3), but the participants showed higher rates of source memory errors when pair-mates shared a parent compared with when they did not [*t*_(39)_ = 4.38, *p* < 0.001, d = 0.69; Bonferroni-corrected alpha level: *p* < 0.0125]. There were no overall differences in source memory across the three types of trained associations (all t′s < 0.5, p′s > 0.6). Importantly, there was a significant pair-mate type × test item type interaction effect [*F*_(3, 117)_ = 3.31, *p* = 0.02, $\eta_{p}^{2}$ = 0.08]. Follow-up paired *t*-tests revealed no differences between shared and no shared parent pair-mates for the trained associations (all t′s <1.2, p′s > 0.25) but increased false memory for having seen the untrained test items (F2-S2) during both study and test when pair-mates shared a parent compared with when they did not [*t*_(39)_ = 2.66, *p* = 0.01, d = 0.42; Bonferroni-corrected alpha level: *p* < 0.0125]. Thus, the physical resemblance between pair-mate faces led to increases in false memories of the source (observed instead of inferred) in addition to the increases in generalization.

**Table 1:** Experiment 1 source memory responses separated by pair-mate and trial type.

	**Response type**			
	**Study only**	**Test only**	**Both study and test**	**Never**
Face 1—Scene 1				
Shared parent	0.06 (0.20)	0.11 (0.27)	**0.78 (0.34)**	0.05 (0.15)
No shared parent	0.10 (0.28)	0.11 (0.27)	**0.76 (0.39)**	0.03 (0.11)
Face 2—Scene 1				
Shared parent	0.06 (0.23)	0.09 (0.25)	**0.79 (0.36)**	0.06 (0.20)
No shared parent	0.08 (0.21)	0.10 (0.23)	**0.74 (0.39)**	0.09 (0.22)
Face 1—Scene 2				
Shared parent	0.04 (0.13)	0.14 (0.25)	**0.76 (0.34)**	0.06 (0.17)
No shared parent	0.05 (0.15)	0.11 (0.24)	**0.80 (0.32)**	0.04 (0.18)
Face 2—Scene 2				
Shared parent	0.06 (0.17)	**0.24 (0.34)**	0.58 (0.37)	0.13 (0.25)
No shared parent	0.06 (0.17)	**0.38 (0.35)**	0.40 (0.41)	0.16 (0.31)
Recombined face	0.04 (0.11)	0.14 (0.20)	0.25 (0.28)	**0.58 (0.32)**
Recombined scenes	0.04 (0.13)	0.06 (0.19)	0.16 (0.23)	**0.73 (0.36)**
Recombined all	0.01 (0.06)	0.07 (0.13)	0.06 (0.14)	**0.86 (0.23)**

*Mean (SD). Bolded rate = correct response for a given trial type*.

Lastly, we tested whether the participants falsely remembered all new pairings on the source memory test as having been presented during both the study and test phase or if this effect was particular to the untrained inference pairs. To do so, we compared the rate of “both” responses for untrained associations to each type of recombined trial, separately for the shared parent pair-mates and no shared parent pair-mates. In all cases, the participants responded “both” to the untrained pairs at numerically higher rates than any type of recombined trial. All comparisons of untrained vs. recombined trials reached Bonferroni corrected threshold of *p* < 0.0083 (t′s > 4.7, p′s < 0.001), with the exception of untrained no shared parent condition compared with the recombined face condition [*t*_(39)_ = 2.24, *p* = 0.03, *d* = 0.35] and untrained no shared parent condition compared with the recombined scene condition [*t*_(39)_ = 2.75, *p* = 0.009, *d* = 0.43] that only reached an uncorrected threshold. Thus, the evidence generally points to false source memories being specific to the inference pairings.

#### Comparing Physical Resemblance Effects in Generalization and False Source Memories

The results showed that greater physical resemblance led to more acquired equivalence as well as more false memories for the source of learning for these associations. We then tested whether the sizes of the effect in generalization and source memory were similar or if one was larger than the other. If acquired equivalence was based mostly or entirely on integration at encoding, then we would expect the two effects to be of a similar size. However, if some acquired equivalence judgments were based on flexible retrieval, memory for the source of these associations might be maintained even in the face of successful generalization, making the effect of physical resemblance smaller in source memory judgments. To test this idea, we computed a 2 (test: acquired equivalence, source memory) × 2 (pair-mate type: shared parent, no shared parent) ANOVA on rates of generalization and false memory for the inference pairs (F2-S2) and were specifically interested in the interaction effect. The test × pair-mate type interaction effect was not significant [*F*_(1, 39)_ = 1.28, *p* = 0.27, $\eta_{p}^{2}$ = 0.03]. Thus, we did not see strong evidence for a differential effect of physical resemblance on generalization and false memory.

### Discussion

In Experiment 1, we tested the hypothesis that greater physical resemblance between pair-mates in an acquired equivalence paradigm would lead to increased generalization across related faces. We also hypothesized that increases in generalization would be accompanied by increases in false memory for the source of generalized associations. The results confirmed both hypotheses. Rates of acquired equivalence were higher when pair-mate faces were blended with a shared parent compared with when pair-mate faces were blended without a shared parent. This difference in generalization for the shared vs. no-shared parent conditions arose despite comparably high performance for the associations presented during training. False memory rates were also higher for the shared parent pair-mates than the no shared parent pair-mates: the participants tended to erroneously believe that the untrained associations had been presented during both the training and test phases rather than in the test phase alone. This difference in source memory for the untrained associations emerged, while source memory for the trained associations did not differ for the shared and no shared parent pair-mates. Together, these findings provide initial evidence that physical resemblance may serve as a cue to reactivate prior experience while encoding related information, leading to integration of the related experiences to support generalization but potentially losing some contextual details.

## Experiment 2

In Experiment 1, we manipulated physical resemblance in a binary manner: pair-mate faces either shared a parent or did not share a parent. Prior study has shown that the degree of similarity, not just its presence or absence, can be an important factor affecting memory fidelity (Turney and Dennis, 2017; Bowman et al., 2019). Graded similarity between items also affects the likelihood of inference across related events, although in a more all-or-none manner (Molitor et al., 2021). However, as these prior studies have not measured memory fidelity and inference across the same events, it is unclear whether graded vs. all-or-none effects of similarity were driven by differences in task parameters across studies or a true divergence across these types of memory judgments. In Experiment 2, we tested how rates of acquired equivalence and false memory for the source of inferred associations varied across levels of pair-mate resemblance that varied parametrically. We constructed pair-mate faces that all shared a parent face but differed in the degree to which the shared parent influenced the final blend (Figure 2B). As in Experiment 1, we tested how likely the participants were to treat the pair-mate faces as equivalent and how often the participants falsely remembered encountering inferred associations during training.

### Materials and Method

#### Participants

Thirty-eight participants from the University of Oregon completed the experiment for course credit. One participant was excluded because of incomplete data, leaving 37 participants reported in all analyses (26 females, mean age = 19.24 years, SD age = 2.41 years, age range = 18–28 years). This sample size was within the range of prior acquired equivalence studies collected in the laboratory (de Araujo Sanchez and Zeithamova, 2020) and was selected, because it would be well-powered to detect a moderate effect size (d = 0.5). We did not have prior data to estimate an effect size across a physical similarity gradient, but data from Experiment 1 suggested that comparisons between individual blend levels (i.e., 50 and 1%) would likely be within this range. All the participants completed written informed consent, and the Institutional Review Board of the University of Oregon approved all procedures.

#### Materials

Blended faces were combined with nature scenes and city scenes to create four quadruplets in a manner similar to Experiment 1. However, rather than having pair-mate faces either share a parent face or not, all pair-mate faces shared a parent face. The pair-mates differed in the level at which the shared parent face was blended: pair-mates with the least overlap between Face 1 and Face 2 had a shared parent blended at 1%, followed by pair-mates with a shared parent blended at 25%, followed by pair-mates with a shared parent blended at 50%, and lastly the pair-mates with the most overlap between Face 1 and Face 2 had a shared parent blended at 75% (Figure 2B). Each blend level was applied to one quadruplet in the set. Besides the difference in the blend procedure, stimulus generation was the same as in Experiment 1 with the exception that there were 20 possible unaltered faces to choose from based on the creation of additional face blends in the time between experiments.

#### Procedure

The overall procedure followed that of Experiment 1 with the addition of a recognition test following the source memory test. Deviations from the Experiment 1 procedure are noted below.

##### Initial Exposure

Rather than being shown in a fixed order, stimulus order was randomized with the constraint that no face was shown twice in a row.

##### Training

The participants underwent six blocks of training, each containing 24 trials for a total of 144 training trials. In each block, the participants saw each of the three trained associations from each of the four quadruplets twice in a random order. The question cues were indicated by “Vacation?” or “Live?” displayed at the top of the screen, and the feedback for trials in which the participants did not respond within the 3 s allotted was changed to “Sorry, too slow!”

##### Acquired Equivalence Test

The acquired equivalence test was as in Experiment 1, except that the question cues at the top of the screen were “Vacation?” and “Live?” as in the training phase of this experiment.

##### Source Memory Test

The source memory test for the trained and untrained associations was as in Experiment 1 with the exception that these associations were tested twice to obtain stable source memory estimates. Differing from Experiment 1, we revised the “recombined scene” condition to match the scene options to be both city or both nature scenes. For recombined conditions, a coding error resulted in some trials being the same as the F2-S2 trials. These trials were excluded from all analyses. Altogether, there were 40 trials in the source memory test.

##### Face Recognition Test

In addition to the acquired equivalence and source memory tests that were of primary interest, we also included a face recognition test. However, it was aimed at testing the discriminability of a face at its different blend levels (e.g., 50 vs. 75%), which was not relevant for the current goals. We, thus, do not discuss this recognition test further.

#### Design and Statistical Analysis

The design and analytical approach were as in Experiment 1 except that there were four levels of physical resemblance rather than two.

### Results

#### Training

Mean accuracies for each type of trained association are presented in Table 2. To test how resemblance between pair-mate faces affected learning, we computed a 4 (shared parent blend level: 1, 25, 50, and 75%) × 3 (trained association type: F1-S1, F2-S1, and F1-S2) × 6 (training block: 1–6) repeated-measures ANOVA. There was a significant main effect of training block [*F*_(2.31, 82.97)_ = 39.75, *p* < 0.001, $\eta_{p}^{2}$ = 0.53, GG] accompanied by a significant linear effect [*F*_(1, 36)_ = 70.88, *p* < 0.001, $\eta_{p}^{2}$ = 0.66] showing increasing accuracy across training. There was also a significant main effect of trained association type [*F*_(2, 72)_ = 4.38, *p* = 0.02, $\eta_{p}^{2}$ = 0.11]. Overall learning was significantly better for F2-S1 pairs (M = 0.75, SD = 0.16) compared with F1-S2 pairs [M = 0.69, SD = 0.16; *t*_(36)_ = 2.68, *p* = 0.011, *d* = 0.44; Bonferroni-corrected alpha level: *p* < 0.016]. No other difference passed the corrected alpha level (t′s <2.2, p′s > 0.04). There was also a significant main effect of pair-mate blend level [*F*_(3, 108)_ = 3.27, *p* = 0.02, $\eta_{p}^{2}$ = 0.08]. Overall, accuracy was numerically poorest for 25% shared parent pair-mates (M = 0.7, SD = 0.17), followed by those with 50% shared parent (M = 0.71, SD = 0.17), then those with 1% shared parent (M = 0.74, SD = 0.17), and those with 75% shared parent had the highest overall accuracy (M = 0.77, SD = 0.17). However, only the difference between 75 and 25% blends reached significance [*t*_(36)_ = 3.09, *p* = 0.004, d = 0.51; all other t′s <2.4, p′s > 0.01; Bonferroni-corrected alpha level: *p* < 0.0083]. No interaction effect reached significance (all F′s <1.8, p′s > 0.1).

**Table 2:** Experiment 2 training accuracy separated by block, shared parent blend level, and trained association type.

**Experiment 2**	**Training block**					
	**1**	**2**	**3**	**4**	**5**	**6**
Face 1—Scene 1						
1%	0.54 (0.34)	0.62 (0.40)	0.78 (0.32)	0.78 (0.34)	0.85 (0.29)	0.88 (0.27)
25%	0.53 (0.37)	0.57 (0.39)	0.66 (0.37)	0.69 (0.43)	0.74 (0.35)	0.84 (0.29)
50%	0.55 (37)	0.73 (0.37)	0.73 (0.37)	0.76 (0.35)	0.76 (0.33)	0.88 (0.25)
75%	0.76 (0.35)	0.76 (0.37)	0.82 (0.32)	0.85 (0.26)	0.87 (0.30)	0.82 (0.34)
Face 2—Scene 1						
1%	0.51 (0.38)	0.69 (0.34)	0.72 (0.32)	0.76 (0.35)	0.88 (0.30)	0.87 (0.30)
25%	0.57 (0.36)	0.53 (0.41)	0.72 (0.38)	0.82 (0.29)	0.92 (0.25)	0.85 (0.26)
50%	0.54 (0.40)	0.61 (0.36)	0.76 (0.33)	0.84 (0.26)	0.81 (0.32)	0.84 (0.29)
75%	0.57 (0.36)	0.72 (0.36)	0.82 (0.34)	0.88 (0.27)	0.84 (0.31)	0.91 (0.20)
Face 1—Scene 2						
1%	0.57 (0.34)	0.72 (0.38)	0.80 (0.36)	0.72 (0.38)	0.77 (0.32)	0.81 (0.32)
25%	0.49 (0.34)	0.57 (0.38)	0.70 (0.34)	0.72 (0.36)	0.80 (0.34)	0.87 (0.28)
50%	0.46 (0.40)	0.60 (0.42)	0.68 (0.34)	0.72 (0.34)	0.77 (0.38)	0.70 (0.38)
75%	0.51 (0.38)	0.60 (0.41)	0.74 (0.33)	0.66 (0.37)	0.78 (0.34)	0.87 (0.28)

*Mean (SD)*.

#### Acquired Equivalence

**Figure 5:**
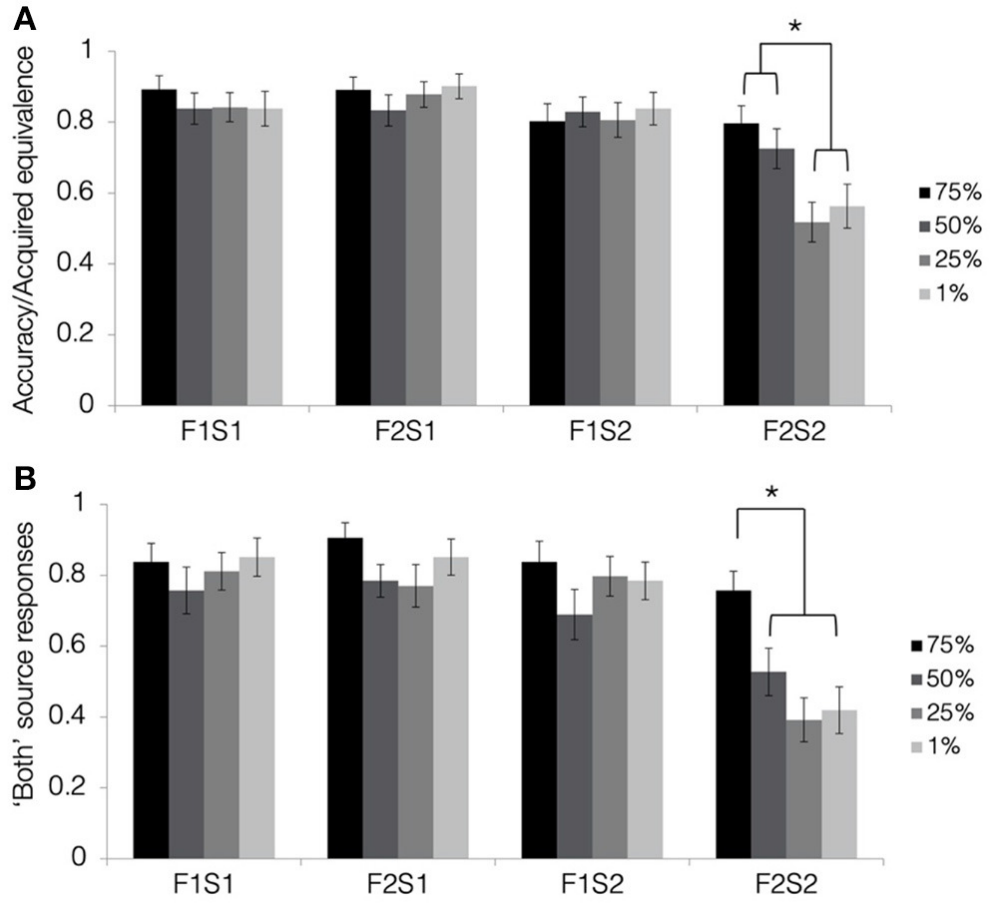
Results from the tests of acquired equivalence and source memory in Experiment 2. **(A)** Acquired equivalence test accuracy for trained associations (F1S1, F2S1, and F1S2) and rates of acquired equivalence for untrained associations (F2S2). **(B)** For the source memory test, the proportion of trials where the participants responded that they had seen the items together during both study and test. This was the correct response for the trained associations but constituted a false memory for the untrained associations, since they were presented only during the test. All results are depicted separately for pair-mates at each blend level. Stars indicate a significant paired difference, following a significant test item type × shared parent blend level interaction effect (alpha = 0.0083).

Figure 5A depicts results from the acquired equivalence test in terms of accuracy for trained associations (F1-S1, F2-S1, and F1-S2) and the proportion of trials showing acquired equivalence for untrained associations (F2-S2). First, we tested whether rates of acquired equivalence differed significantly from chance using a one-sample *t*-test at each blend level. Results revealed that rates of acquired equivalence differed from chance for pair-mates blended at 75 and 50% (both t′s > 4, p′s < 0.001; Bonferroni-corrected alpha-level: *p* < 0.0125) but not those blended at 25 or 1% (both t′s <1.1, p′s > 0.31).

To test how resemblance between pair-mate faces affected the tendency to show acquired equivalence, we computed a 4 (shared parent blend level: 1, 25, 50, and 75%) × 4 (test item type: F1-S1, F2-S1, F1-S2, and F2-S2) repeated-measures ANOVA. There was a significant main effect of test item type [*F*_(2.14, 77.08)_ = 33.9, *p* < 0.001, $\eta_{p}^{2}$ = 0.49, GG], with F2-S1 pairs having the highest numeric accuracy (M = 0.88, SD = 0.19), followed by F1-S1 pairs (M = 0.85, SD = 0.21), and F1-S2 pairs (M = 0.82, SD = 0.23), and with F2-S2 pairs showing the lowest accuracy (M = 0.65, SD = 0.21). Accuracy for all types of trained associations was significantly higher than for untrained associations (all t′s > 5, p′s < 0.001; Bonferroni-corrected alpha level: *p* < 0.0083). Differences between the trained associations did not pass correction for multiple comparisons (t′s <2.8, p′s > 0.009). There was also a significant main effect of shared parent blend level [*F*_(3, 108)_ = 3.58, *p* = 0.02, $\eta_{p}^{2}$ = 0.09]. Overall accuracy was highest for pair-mates with a shared parent blended at 75% (M = 0.85, SD = 0.24), followed by those blended at 50% (M = 0.81, SD = 0.21), those blended at 1% (M = 0.79, SD = 0.2), and lastly those blended at 25% (M = 0.76, SD = 0.21). Those blended at 75% differed significantly from those blended at 25% [*t*_(36)_ = 3.75, *p* = 0.001, d = 0.62; Bonferroni-corrected alpha level: *p* < 0.0083]. No other pairwise comparisons passed a correction for multiple comparisons (all t′s <2.2, p′s > 0.03).

Lastly, there was a significant test item type × blend level interaction effect [*F*_(5.85, 210.75)_ = 3.9, *p* = 0.001, $\eta_{p}^{2}$ = 0.1, GG]. To determine the nature of this interaction, we computed a separate one-way ANOVA for each test item type, testing for an effect of the blend level. Accuracy for the trained associations did not differ significantly based on the percentage of the shared parent making up the faces (all F′s <2, p′s > 0.2), but the blend level did affect rates of acquired equivalence for the untrained associations (i.e., F2-S2 pairs) [*F*_(3, 108)_ = 6.73, *p* < 0.001, $\eta_{p}^{2}$ = 0.16; Bonferroni-corrected alpha level: *p* < 0.0125]. Rates of acquired equivalence were highest for pair-mates with the shared parent blended at 75% (M = 0.8, SD = 0.3), followed by those blended at 50% (M = 0.73, SD = 0.34), those blended at 1% (M = 0.56, SD = 0.38), and lastly those blended at 25% (M = 0.52, SD = 0.34). Rates of acquired equivalence for the two highest blend levels (75 and 50%) differed significantly from the two lowest blend levels (1 and 25%; all t′s > 3, p′s < 0.003; Bonferroni-corrected alpha level: *p* < 0.0083) but did not differ significantly from one another [*t*_(36)_ = 1.31, *p* = 0.2, d = 0.22]. Rates of acquired equivalence for the two lowest blend levels likewise did not differ significantly from one another [*t*_(36)_ = −0.56, *p* = 0.58, d = 0.09]. Thus, the similarity of items across related trials affected generalization but not memory for trained associations. Despite the apparent discontinuity in rates of generalization across blend levels, only the linear effect of blend level reached significance [*F*_(1, 36)_ = 13.43, *p* = 0.001, $\eta_{p}^{2}$ = 0.27]. Neither the quadratic effect [*F*_(1, 36)_ = 1.48, *p* = 0.23, $\eta_{p}^{2}$ = 0.04] nor the cubic effect reached significance [*F*_(1, 36)_ = 3.15, *p* = 0.08, $\eta_{p}^{2}$ = 0.08].

#### Source Memory Test

Mean response rates for all source memory response options are presented in Table 3. Mean proportion of “both study and test” responses for each pair and quadruplet type are presented in Figure 5B. To test whether the participants tended to falsely remember untrained pairs as having been presented during both the study and test phase, we computed a 4 (shared parent blend level: 1, 25, 50, and 75%) × 4 (test item type: F1-S1, F2-S1, F1-S2, and F2-S2) repeated-measures ANOVA on the proportion of “both study and test” responses. There was a significant main effect of test item type [*F*_(3, 108)_ = 33.8, *p* < 0.001, $\eta_{p}^{2}$ = 0.09], with higher rates of “both study and test” responses for trained associations (F1-S1 M = 0.81, SD = 0.21; F2-S1 M = 0.83, SD = 0.19; F1-S2 M = 0.78, SD = 0.23) compared with untrained associations (F2-S2 M = 0.52, SD = 0.21; all t′s > 6, p′s < 0.001; Bonferroni-corrected alpha level: *p* < 0.0083). No differences between trained associations reached significance (all t′s <1.95, p′s > 0.06). There was also a significant main effect of shared parent blend level [*F*_(3, 108)_ = 5.03, *p* = 0.003, $\eta_{p}^{2}$ = 0.12]. Numerically, items with the parent face blended at 75% were most likely to be judged as having been presented during both study and test (M = 0.83, SD = 0.24), followed by those blended at 1% (M = 0.73, SD = 0.23), those blended at 25% (M = 0.69, SD = 0.21), and lastly those blended at 50% (M = 0.69, SD = 0.25). Rates of “both” responses were significantly higher for 75% blends compared with 50% blends and 25% blends (both t′s > 2.9, p′s = 0.005; Bonferroni-corrected alpha level: *p* < 0.0083), and no other difference between blend levels passed the corrected alpha level (all t′s <2.5, p′s > 0.02).

**Table 3:** Experiment 2 source memory responses separated by shared parent blend level and trial type.

	**Response type**			
	**Study only**	**Test only**	**Both study and test**	**Never**
Face 1—Scene 1				
1%	0.04 (0.14)	0.07 (0.21)	**0.85 (0.33)**	0.04 (0.18)
25%	0.04 (0.14)	0.12 (0.27)	**0.81 (0.32)**	0.03 (0.11)
50%	0.05 (0.20)	0.14 (0.30)	**0.76 (0.40)**	0.05 (0.20)
75%	00 (0.00)	0.12 (0.27)	**0.84 (0.31)**	0.04 (0.14)
Face 2—Scene 1				
1%	0.03 (0.11)	0.08 (0.22)	**0.85 (0.31)**	0.04 (0.18)
25%	0.04 (0.14)	0.12 (0.30)	**0.77 (0.37)**	0.07 (0.21)
50%	0.01 (0.08)	0.16 (0.26)	**0.78 (0.28)**	0.04 (0.14)
75%	0.04 (0.14)	0.05 (0.20)	**0.91 (0.26)**	0.00 (0.00)
Face 1—Scene 2				
1%	0.01 (0.08)	0.12 (0.25)	**0.78 (0.32)**	0.08 (0.19)
25%	0.03 (0.11)	0.10 (0.20)	**0.80 (0.34)**	0.08 (0.22)
50%	0.04 (0.18)	0.16 (0.33)	**0.69 (0.43)**	0.11 (0.27)
75%	0.01 (0.08)	0.11 (0.29)	**0.84 (0.35)**	0.04 (0.14)
Face 2—Scene 2				
1%	0.04 (0.14)	**0.37 (0.42)**	0.42 (0.40)	0.18 (0.32)
25%	0.04 (0.14)	**0.45 (0.40)**	0.39 (0.38)	0.12 (0.27)
50%	0.04 (0.14)	**0.30 (0.40)**	0.53 (0.41)	0.14 (0.25)
75%	0.03 (0.11)	**0.16 (0.29)**	0.76 (0.33)	0.05 (0.16)
Recombined face	0.05(0.15)	0.14(0.22)	0.22(0.33)	**0.59 (0.36)**
Recombined scene	0.05 (0.11)	0.11 (0.15)	0.21 (0.25)	**0.63 (0.34)**

*Mean (SD). Bold font = correct response for a given trial type*.

Lastly, there was a significant test item type × blend level interaction effect [*F*_(9, 324)_ = 2.49, *p* = 0.009, $\eta_{p}^{2}$ = 0.07]. To understand the nature of this interaction, we computed a separate one-way ANOVA for each test item type, looking for an effect of blend level on rates of “both” responses. The effect of blend level did not reach significance for any of the trained association types (all F′s <2, p′s > 0.12), but there was a significant effect of blend level for the untrained association [*F*_(3, 108)_ = 7.59, *p* < 0.001, $\eta_{p}^{2}$ = 0.17; Bonferroni-corrected alpha level: *p* < 0.0125]. The participants were most likely to falsely remember having seen the untrained associations during the study when they were blended at 75% shared parent (M = 0.76, SD = 0.33), and this false memory rate was higher than for all the other blend levels (50% M = 0.53, SD = 0.41; 25% = 0.39, SD = 0.38; 1% M = 0.42, SD = 0.4; all t′s > 2.9, p′s < 0.007; Bonferroni-corrected alpha level: *p* < 0.0083). There were no significant differences among the remaining blend levels (all t′s <1.7, p′s > 0.1). Thus, the similarity between pair-mate faces did not affect source memory for the trained associations, but the highest level of similarity led the participants to falsely remember having seen the untrained association during the study.

#### Comparing Physical Resemblance Effects in Generalization and False Source Memories

As in Experiment 1, we were interested in whether the effect of physical resemblance was of a similar magnitude in acquired equivalence and source false memories. Qualitatively, the pattern in Experiment 2 was different for 50% blends than it was in Experiment 1 shared parent condition as increased generalization in that condition was not accompanied by increased false memory when compared with the low similarity condition. To test for potential differential effects of similarity on the two measures formally, we computed a test (acquired equivalence, source memory) × shared parent blend level (1, 25, 50, and 75%) ANOVA on rates of generalization and false memory for inference pairs (F2-S2). As in Experiment 1, the interaction effect was not significant [*F*_(3, 108)_ = 0.88, *p* = 0.46, $\eta_{p}^{2}$ = 0.02]. Thus, physical resemblance had a similar effect on generalization and source memory when all blend levels were considered.

### Discussion

In Experiment 2, we tested whether parametrically increasing similarity across pair-mates in an acquired equivalence paradigm would lead to corresponding increases in rates of generalization and false memories for the source of inferred associations. We reasoned that resemblance can serve as a cue to reactivate prior experiences, leading to their integration. Integration across related episodes could then facilitate generalization but could lead the participants to confuse the source of inferred associations. The results were mostly but not fully consistent with this idea. Consistent with the hypothesis and with Experiment 1, we found that higher degrees of pair-mate resemblance were associated with higher rates of acquired equivalence as well as a greater tendency to falsely remember having seen untrained associations during the training phase. The effect of similarity level on generalization and false memory emerged for the untrained associations despite similarly high performance across different levels of pair-mate similarity for the trained associations. However, unlike in Experiment 1, we did not find increased source memory confusion in the 50% blend condition compared with the 1% blend condition, even though we found the levels of generalization in that condition were well above chance and significantly higher than in the 1% blend condition. Thus, whether similarity level affects generalization and source memory equally was somewhat inconclusive in Experiment 2. Finally, we noted that in the 75% blend condition, the participants endorsed untrained F2-S2 pairs as being seen at both study and test to a high level that was approaching the rate of endorsing the actually studied F1-S2 pairs (proportion of 0.78 vs. 0.84). Thus, we wanted to test an alternative explanation of the results from the 75% blend condition: that the participants simply could not tell pair-mate faces apart at the higher levels of physical resemblance. If this was the case, it might appear that the participants were actively generalizing across the two faces when, in fact, they simply did not notice that there were two faces rather than only one. We, thus, ran a follow-up experiment to determine the degree to which pair-mate faces at each level of resemblance were distinguishable from one another as well as gather additional data to further test whether the effect of similarity on generalization and source memory confusion go hand in hand.

## Experiment 3

**Figure 6:**
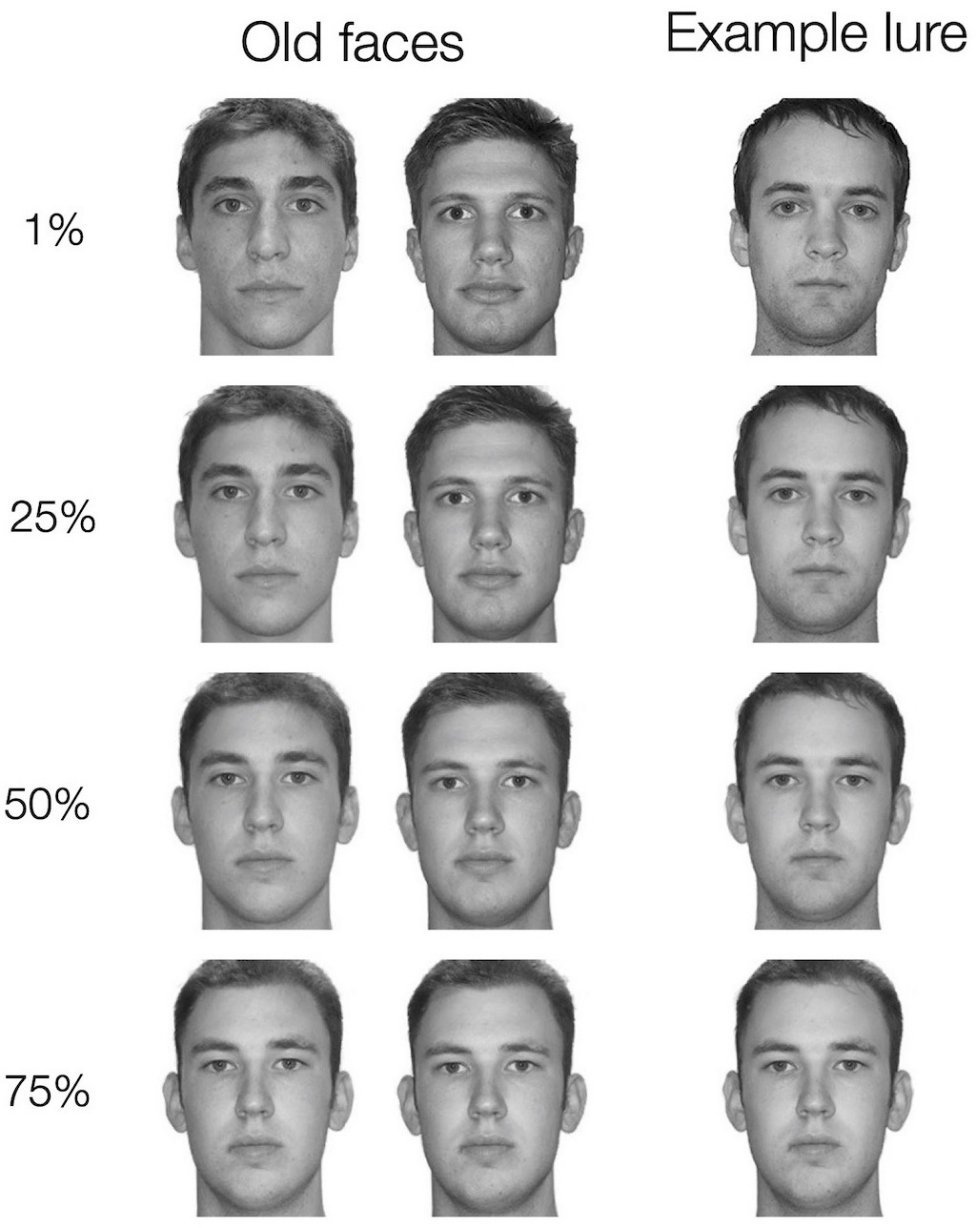
Example recognition stimuli for Experiment 3. Faces in the first two columns represent an example pairmate from the training phase, which served as old faces during the recognition test. The second column represents an example new (lure) face. Each row shows the example pairmate at corresponding lure at a different blend level with the lure always matching the blend level of the associated pairmate. During the experiment, each pairmate and lure would be shown at one blend level. All the faces depicted come from the Dallas Face Database (Minear and Park, 2004).

Experiment 3 followed the overall design of Experiment 2 except that we included a face recognition test that immediately followed the training phase. After the recognition test, the participants completed the acquired equivalence test and the source memory test as in prior experiments. The recognition test included the old training faces as well as lures that were blends of the shared parent faces from training with new parent faces that were not used to generate the training sets (see Figure 6). We tested recognition prior to acquired equivalence and source memory, so that we would have the best possible measure of the ability to discriminate faces during learning, as there is evidence that generalization itself can reduce subsequent memory specificity (Carpenter and Schacter, 2017, 2018). However, this design feature makes the acquired equivalence and source memory data less comparable with Experiment 2 since performing a recognition task could also affect these other types of memory judgments. Nonetheless, it served the primary goals of measuring the discriminability of pair-mate faces at each blend level, as well as testing the common or differential effects of similarity level on generalization and source memory.

### Materials and Method

#### Participants

Prior to data collection, the sample size was determined based on the effect of blend level on generalization from Experiment 2 ($\eta_{p}^{2}$ = 0.16), which was the smallest of all observed effect sizes for the main effects of interest (similarity level effects on generalization and source memory) across Experiments 1 and 2. Using the repeated measures ANOVA protocol from GPower (Faul et al., 2007), we determined that *N* = 33 was a sufficient sample to obtain 80% power for an effect of this size. We recruited subjects from the University of Oregon who completed the experiment online for course credit. A total of 37 participants were recruited in order to have 33 subjects retained in all analyses. Four subjects were excluded and replaced because of poor learning during the training phase (<60% overall accuracy in the last block of training). Demographic data for six participants were lost because of experimenter error, leaving reportable demographics for 27 participants (13 females, mean age = 20.2 years, SD age = 3.1 years, age range = 18–34 years). Although demographics were missing for some subjects, we retained those subjects for the experimental task, leaving data from 33 subjects reported in all analyses. All the participants were provided with the consent information and asked to affirm it with a button press. The Institutional Review Board of the University of Oregon approved all procedures.

#### Materials

The stimulus sets were constructed as in Experiment 2 with the exception that, rather than creating a new randomization of parent faces and scene assignment for each participant, three randomized sets were created and counterbalanced across the participants.

#### Procedure

Data for Experiment 3 were collected online through pavlovia.org. The overall procedure followed that of Experiment 2 except that the recognition test followed immediately after training rather than following the source memory test. This change ensured that any effects of blend level on recognition were not due to demand-driven integration during the generalization test (Carpenter and Schacter, 2017, 2018). While there were just three possible stimulus sets that were counterbalanced across the participants, the presentation order within each phase was randomized for each participant. Deviations from the Experiment 2 procedure for specific tasks are noted below.

##### Initial Exposure

Faces were shown in a random order for each of the three repetitions. It was possible for a face to be shown twice in a row only if a given face was the last one shown in one repetition and the first one shown in the following repetition.

##### Training

The question cues were indicated as in Experiment 1: “Where does he live?” or “Where does he vacation?”. The feedback for trials in which the participants did not respond within the 3 s allotted was “Too slow.”

##### Face Recognition Test

Immediately following training, the participants completed an old/new face recognition test. Figure 6 depicts an example face recognition set. The old faces were the eight ones presented during the training phase—two pair-mate faces at each blend level. We generated lure faces by taking the eight unblended parent faces that remained after generating the training set and assigning each of them to be blended with the shared parent face of one set of pair-mates. This led to two lures at each blend level for a total of eight new items at recognition. The new unblended faces were blended with the shared parent face at the same blend level as the original pair-mates. For example, the lures for the pair-mates blended with 1% shared parent were the new unblended faces blended with the shared parent also at 1%. This allowed us to test whether the participants could distinguish between faces made up of a given shared parent at the blend level experienced during training. If they were able do so for new lures, then they were likely able to distinguish between the two pair-mate faces from the training set as well.

Each face was presented once during the recognition phase. Each face was presented for 3 s during which time the participants could make their old/new response. This timing matched the length of face presentation and response time from the training phase. Each face was followed by a 1-s fixation cross.

##### Acquired Equivalence Test

The acquired equivalence test was as in Experiment 2, except that the question cues at the top of the screen were as in the training phase of this experiment.

##### Source Memory Test

The source memory test was as in Experiment 2 with two exceptions. First, associations were tested only once. Second, the recombined face condition consisted of two scenes that had been together as alternative choices but with a different face. This condition was always distinct from the F2-S2 condition. The recombined scene condition consisted of a face with its target scene but with a new distractor. The distractor always came from the same category (city scene, nature scene) as the target.

#### Design and Statistical Analysis

The design and analytical approach were as in Experiment 2, except that there was an additional dependent variable of interest: recognition scores that indicated the ability to distinguish training faces from new faces using the same shared parent face at the same blend level.

### Results

#### Training

Mean accuracies for each type of trained association are presented in Table 4. Consistent with Experiments 1 and 2, there was a significant main effect of training block [*F*_(3.1, 99.1)_ = 54.85, *p* < 0.001, $\eta_{p}^{2}$ = 0.63, GG] accompanied by a significant linear effect [*F*_(1, 32)_ = 214.52, *p* < 0.001, $\eta_{p}^{2}$ = 0.87] showing increasing accuracy across training. Neither the main effect of trained association type nor the main effect of blend level was significant (both F′s <1.4, p′s > 0.27). There was, however, a significant trained association type × blend level interaction effect [*F*_(6, 192)_ = 3.16, *p* = 0.006, $\eta_{p}^{2}$ = 0.09]. To better understand the nature of this interaction, we computed separate one-way ANOVAs for each type of trained association collapsed across training blocks, testing whether there was an overall effect of the blend level for each. The effect of blend level was only significant for F1-S2 pairs [*F*_(3, 96)_ = 4.61, *p* = 0.005, $\eta_{p}^{2}$ = 0.13; other F′s <1.9, p′s > 0.14]. Training accuracy for the pair-mates blended with 75% shared parent was poorer than all the other pairs (t′s > 3, p′s < 0.005), although the difference from the 1% blend level did not pass a correction for multiple comparisons [*t*_(32)_ = 2.56, *p* = 0.016, *d* = 0.26; Bonferroni corrected alpha level: *p* < 0.0083]. No other pairwise difference was significant (all t′s < 0.5, p′s > 0.7). Thus, blend level did not strongly affect learning for F1-S1 or F2-S1 associations, but there was some evidence of poorer learning for F1-S2 associations when there was a 75% shared parent. No other interaction effect reached significance (all F′s <1.5, p′s > 0.06).

**Table 4:** Experiment 3 training accuracy separated by block, shared parent blend level, and trained association type.

	**Training block**					
	**1**	**2**	**3**	**4**	**5**	**6**
Face 1—Scene 1						
1%	0.52 (0.40)	0.68 (0.39)	0.79 (0.35)	0.78 (0.35)	0.84 (0.31)	0.91 (0.20)
25%	0.55 (0.38)	0.64 (0.31)	0.58 (0.40)	0.71 (0.36)	0.77 (0.40)	0.81 (0.33)
50%	0.50 (0.33)	0.68 (0.35)	0.65 (0.42)	0.71 (0.40)	0.72 (0.37)	0.91 (0.23)
75%	0.49 (0.39)	0.62 (0.42)	0.82 (0.30)	0.74 (0.36)	0.86 (0.31)	0.95 (0.19)
Face 2—Scene 1						
1%	0.55 (0.36)	0.74 (0.38)	0.68 (0.41)	0.75 (0.35)	0.87 (0.31)	0.88 (0.22)
25%	0.58 (0.38)	0.65 (0.40)	0.73 (0.33)	0.71 (0.33)	0.79 (0.31)	0.85 (0.30)
50%	0.50 (0.40)	0.70 (0.33)	0.67 (0.37)	0.80 (0.28)	0.82 (0.33)	0.82 (0.33)
75%	0.56 (0.32)	0.62 (0.42)	0.73 (0.36)	0.85 (0.26)	0.78 (0.36)	0.91 (0.26)
Face 1—Scene 2						
1%	0.59 (0.44)	0.68 (0.33)	0.73 (0.38)	0.82 (0.32)	0.82 (0.32)	0.96 (0.15)
25%	0.42 (0.40)	0.73 (0.36)	0.86 (0.29)	0.82 (0.30)	0.87 (0.28)	0.99 (0.09)
50%	0.64 (0.31)	0.74 (0.33)	0.76 (0.28)	0.77 (0.33)	0.92 (0.22)	0.85 (0.31)
75%	0.53 (0.35)	0.49 (0.34)	0.61 (0.45)	0.70 (0.35)	0.72 (0.39)	0.86 (0.29)

*Mean (SD)*.

#### Face Recognition

**Figure 7:**
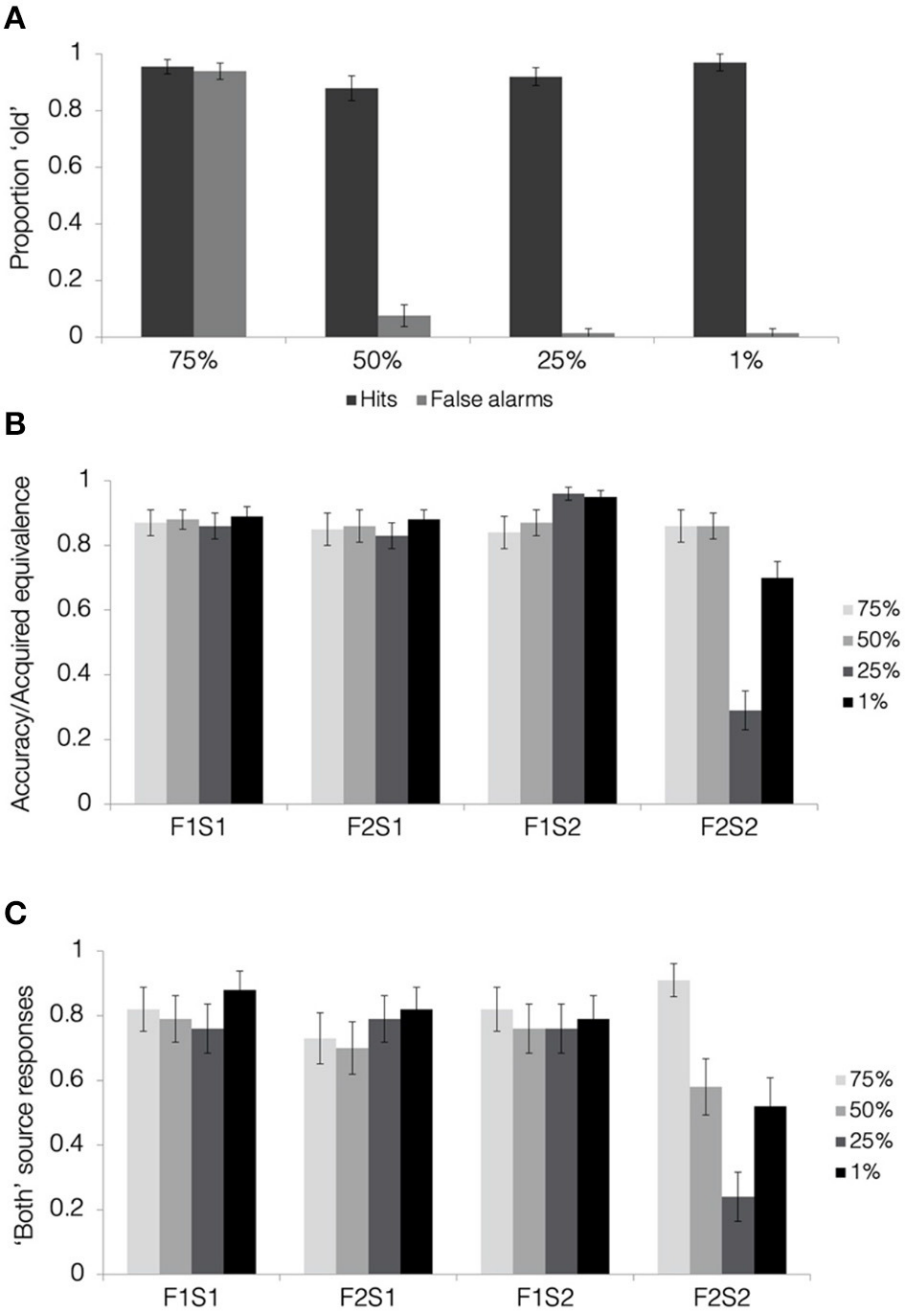
Results from face recognition, acquired equivalence, and source memory tests in Experiment 3. **(A)** Hit and false alarm rates from the face recognition test. **(B)** Acquired equivalence test accuracy for trained associations (F1S1, F2S1, and F1S2) and rates of acquired equivalence for untrained associations (F2S2). **(C)** For the source memory test, the proportion of trials where the participants responded that they had seen the items together during both study and test. This was the correct response for the trained associations but constituted a false memory for the untrained associations, since they were presented only during the test. All results are depicted separately for pair-mates at each blend level. Error bars represent the standard error of the mean across subjects.

Hit and false alarm rates separated by blend level are presented in Figure 7A. Face recognition scores were calculated as the corrected hit rate (hit rate—false alarm rate) separately for each blend level. We first tested whether the participants were able to discriminate old faces from lure faces at above-chance levels for each of the four blend levels. One-sample *t*-tests comparing corrected hit rates with zero (i.e., no old/new discrimination) showed above-chance performance for 1, 25, and 50% blends (all t′s > 14, p′s < 0.001; Bonferroni corrected alpha level: *p* < 0.0125), with all of these conditions showing hit rates above 85% and false alarm rates below 10%. In contrast, corrected hit rates for the 75% blends were not different from chance [*t*_(32)_ = 0.44, *p* = 0.66, *d* = 1.33], driven by a false alarm rate of over 90%. Comparing the corrected hit rates with a one-way, repeated measures ANOVA, we found a significant effect of blend level [*F*_(1.9, 60.3)_ = 140.51, *p* < 0.001, $\eta_{p}^{2}$ = 0.82]. This overall effect was qualified by significant linear [*F*_(1, 32)_ = 642.22, *p* < 0.001, $\eta_{p}^{2}$ = 0.95], quadratic [*F*_(1, 32)_ = 52.63, *p* < 0.001, $\eta_{p}^{2}$ = 0.62], and cubic effects [*F*_(1, 32)_ = 22.01, *p* < 0.001, $\eta_{p}^{2}$ = 0.41]. Pairwise comparisons revealed significantly poorer recognition scores for 75% blends compared with all others (all t′s > 11, p′s < 0.001). Recognition scores for 50% blends were poorer than those for 1% blends [*t*_(32)_ = 2.39, *p* = 0.023, *d* = 0.37] and 25% blends [*t*_(32)_ = 2.51, *p* = 0.017, *d* = 0.25], but these differences did not pass a correction for multiple comparisons (Bonferroni corrected alpha level: *p* < 0.0083).

To summarize, we see clear evidence that the participants were not able to discriminate between faces at the highest degree of physical similarity at 75% blend level. This indicates that the high generalization and high false memory found in Experiment 2 for 75% blends was likely driven by a failure to discriminate between pair-mate faces, simply confusing Face 2 for Face 1. For the rest of this report, we will report results from all the four blend levels in tables and figures for completeness, but we will not consider the 75% blend condition in further analyses. Importantly, we also see clear evidence that the participants were able to distinguish between faces at the other three of the blend levels, 1, 25, and 50%. Thus, increased generalization between pair-mates at 50% blend level observed in Experiments 1 and 2 was unlikely driven by a lack of discrimination between them.

#### Acquired Equivalence

Figure 7B presents rates results from the acquired equivalence test in terms of accuracy for trained associations (F1-S1, F2-S1, and F1-S2) and the proportion of trials showing acquired equivalence for untrained associations (F2-S2). First, we tested whether rates of acquired equivalence differed significantly from chance (0.5) using a one-sample *t*-test at each blend level. Results revealed that rates of acquired equivalence were significantly higher than chance for pair-mates blended at 1 and 50% (all t′s >3.5, p′s < 0.002; Bonferroni-corrected alpha level: *p* < 0.0167). Those blended at 25% showed significantly *below chance* acquired equivalence [*t*_(32)_ = −3.46, *p* = 0.002, *d* = 0.36]. In other words, the participants were more likely to select a scene for Face 2 that was *not* associated with their pair-mate when the pair-mate was at 25% shared level of similarity.

Comparing across association types and blend levels, there was a significant main effect of association type [*F*_(2.5, 79.7)_ = 44.43, *p* < 0.001, $\eta_{p}^{2}$ = 0.58, GG]. Rates of acquired equivalence for the F2-S2 pairs were lower than rates of correct associative memory for all the trained pairs (all t′s >7.1, p′s < 0.001; Bonferroni-corrected alpha level: *p* < 0.0083). Pairwise differences among trained associations (F1-S1, F2-S1, and F1-S2) did not reach significance following correction for multiple comparisons (all t′s <2.5, p′s > 0.02). There was also a main effect of blend level [*F*_(2, 64)_ = 9.94, *p* < 0.001, $\eta_{p}^{2}$ = 0.24]. Scores were lower for pair-mates blended with 25% shared parent compared with all other blend levels (both t′s > 3.7, p′s < 0.002; Bonferroni-corrected alpha level: *p* < 0.0167). The difference between 1 and 50% blends was not significant [*t*_(32)_ = 0.41, *p* = 0.68, *d* = 0.2].

Critically, we found a significant association type × blend level interaction effect [*F*_(3.6, 114)_ = 19.15, *p* < 0.001, $\eta_{p}^{2}$ = 0.37, GG]. To understand the nature of this interaction, we computed separate one-way ANOVAs for each association type, looking for an effect of blend level. The effect of blend level was not significant for any of the trained associations following a correction for multiple comparisons (all F′s <3.4, p′s > 0.04; Bonferroni-corrected alpha level: *p* < 0.0125). There was, however, a significant effect of blend level in acquired equivalence (i.e., F2-S2 pairs) [*F*_(1.8, 57.2)_ = 28.23, *p* < 0.001,$\eta_{p}^{2}$ = 0.47, GG]. Rates of acquired equivalence were higher for 50% blends compared with 1% blends [*t*_(32)_ = 2.55, *p* = 0.016, *d* = 0.37; Bonferroni-corrected alpha level: *p* < 0.0167] and 25% blends [*t*_(32)_ = 6.7, *p* < 0.001, *d* = 0.5]. Rates of acquired equivalence were also higher for 1% blends compared with 25% blends [*t*_(32)_ = 4.85, *p* < 0.001, *d* = 0.49]. The low rates of acquired equivalence for 25% blends was unexpected and not seen in Experiment 2. The exact reason for the low generalization in this condition are not immediately clear. Nonetheless, we replicate the key finding from Experiment 2 that rates of acquired equivalence are highest for 50% compared with the lower 1 and 25% blend levels.

Another way to think about the association type × blend level interaction is to ask whether rates of acquired equivalence matched accuracy rates for trained associations at each blend level. We know that, overall, rates of acquired equivalence were lower than rates of memory for trained associations, but is that true across levels of physical resemblance? Comparing across association types for each blend level, we find lower rates of acquired equivalence compared with trained accuracy for 1% blends and 25% blends (both F′s > 11.4, p′s < 0.001). However, there was no effect of association type for the 50% blends [*F*_(2.5, 80.4)_ = 0.16, *p* = 0.89, $\eta_{p}^{2}$ = 0.005, GG], indicating that the participants chose the acquired equivalence response for untrained pairs to a comparable degree as they remembered the trained associations. This further demonstrates the very robust generalization for the 50% blends.

Lastly, we compared rates of acquired equivalence to rates of false face recognition across blend levels, testing the degree to which differences in generalization track differences in false recognition of faces. We were interested specifically in the test × blend level interaction effect, which was significant [*F*_(2, 64)_ = 22.25, *p* < 0.001, $\eta_{p}^{2}$ = 0.41]. This pattern was driven by rates of acquired equivalence that increased from 25 to 1 to 50% blends (see stats above), whereas mean false alarm rates for lure faces were the same for 1 and 25% blends (M = 1.5%, SD = 8.7%) and only slightly higher for 50% blends (M = 7.6%, SD = 22%). Thus, the patterns in generalization and false recognition of faces did not mirror one another.

#### Source Memory

Mean response rates for all source memory response options are presented in Table 5. Mean proportion of “both study and test” responses for each test item type and each blend level is presented in Figure 7C. Comparing rates of “both” responses across test item types (F1-S1, F2-S1, F1-S2, and F2-S2), and blend levels (1, 25, and 50%) using repeated measures ANOVA, there was a significant main effect of test item type [*F*_(2.2, 69.5)_ = 21.03, *p* < 0.001, $\eta_{p}^{2}$ = 0.4, GG]. Rates of “both study and test” responses were higher for all trained associations (for which it was the correct response) compared with untrained associations (all t′s > 5, p′s < 0.001; Bonferroni-corrected alpha level: *p* < 0.0083) when collapsed across blend levels. Pairwise differences among trained associations were not significant (all t′s <1.2, p′s > 0.2). The main effect of blend level was not significant [*F*_(1.6, 51.7)_ = 2, *p* = 0.14, $\eta_{p}^{2}$ = 0.06, GG]. The test item type × blend level interaction was also not significant [*F*_(4, 129.5)_ = 2.15, *p* = 0.078, $\eta_{p}^{2}$ = 0.06]. Although this interaction was significant in Experiment 2, it was driven by higher rates of source errors for 75% blends compared with all others. Ignoring that condition in which the faces were not discriminable from one another in memory, Experiments 2–3 show little effect of the blend level on source memory judgments.

**Table 5:** Experiment 3 source memory responses separated by shared parent blend level and trial type.

	**Response type**			
	**Study only**	**Test only**	**Both study and test**	**Never**
Face 1—Scene 1				
1%	0.03 (0.17)	0.03 (0.17)	**0.88 (0.33)**	0.06 (0.24)
25%	0.12 (0.33)	0.03 (0.17)	**0.76 (0.44)**	0.09 (0.29)
50%	0.06 (0.24)	0.06 (0.24)	**0.79 (0.42)**	0.09 (0.29)
75%	0.06 (0.24)	0.06 (0.24)	**0.82 (0.39)**	0.06 (0.24)
Face 2—Scene 1				
1%	0.06 (0.24)	0.09 (0.29)	**0.82 (0.39)**	0.03 (0.17)
25%	0.06 (0.24)	0.06 (0.24)	**0.79 (0.42)**	0.09 (0.29)
50%	0.03 (0.17)	0.18 (0.39)	**0.70 (0.47)**	0.09 (0.29)
75%	0.09 (0.29)	0.09 (0.29)	**0.73 (0.45)**	0.09 (0.29)
Face 1—Scene 2				
1%	0.06 (0.24)	0.12 (0.33)	**0.79 (0.42)**	0.03 (0.17)
25%	0.03 (0.17)	0.09 (0.29)	**0.76 (0.44)**	0.12 (0.33)
50%	0.03 (0.17)	0.09 (0.29)	**0.76 (0.44)**	0.12 (0.33)
75%	0.00 (0.00)	0.09 (0.29)	**0.82 (0.39)**	0.09 (0.29)
Face 2—Scene 2				
1%	0.09 (0.29)	**0.21 (0.42)**	0.52 (0.51)	0.18 (0.39)
25%	0.12 (0.33)	**0.48 (0.51)**	0.24 (0.44)	0.15 (0.36)
50%	0.03 (0.17)	**0.30 (0.47)**	0.58 (0.50)	0.09 (0.29)
75%	0.03 (0.17)	**0.00 (0.00)**	0.91 (0.29)	0.06 (0.24)
Recombined face	0.06 (0.11)	0.13 (0.13)	0.23 (0.26)	**0.58 (0.35)**
Recombined scene	0.10 (0.18)	0.06 (0.09)	0.28 (0.22)	**0.56 (0.32)**

*Mean (SD) Bold font = correct response for a given trial type*.

#### Comparing Physical Resemblance Effects in Generalization and False Source Memories

As in Experiments 1–2, we compared blend level effects for generalization and source memory to determine the degree to which physical resemblance had a similar or different effect in generalization and source memory. As in prior experiments, the test × blend level interaction effect was not significant [*F*_(2, 64)_ = 1.46, *p* = 0.24,$\eta_{p}^{2}$ = 0.04]. Thus, although there was a significant effect of blend level on generalization but not source memory errors, the overall patterns did not differ reliably across tests.

### Discussion

The aim of Experiment 3 was to test whether the effect of physical resemblance on generalization and source memory could be explained by the poor discriminability of pair-mate faces at higher blend levels. Poor discriminability was clear for 75% blends, which were not reliably discriminable from one another in memory. Thus, source memory and generalization results for this condition were likely driven purely by the confusability of the pair-mate faces. In contrast, we replicated high rates of generalization for 50% blends while demonstrating that faces blended at 50% shared parent retained good discriminability. Interestingly, generalization rates for 50% blends in Experiment 3 were comparable with memory for trained relationships in this condition (86% generalization vs. 87% trained accuracy). Furthermore, the results indicate that increased similarity does not have to lead to source memory confusion, at least not at the 50% blend level where discriminability is maintained. Overall, results from Experiment 3 bolster the idea that physical resemblance between related experiences can foster generalization. The results also indicate that better generalization is not always associated with tradeoffs in terms of increased false memories.

## General Discussion

In this study, we manipulated the degree of resemblance between items constituting related experiences and tested both the tendency to generalize across those experiences and the specificity of memory for separate experiences. We predicted that higher degrees of overlap would make it more likely that the participants would reactivate the prior related episode when encountering new, related information and, therefore, increase the likelihood that representations of those episodes would become integrated. As a signature of memory integration, we expected higher rates of generalization accompanied by a source memory confusion, such as mistaking the inferred information for a directly observed one. We found partial support for this hypothesis. Rates of generalization (acquired equivalence) were higher when there was more physical resemblance across related episodes, suggesting that physical resemblance helped the participants make links across experiences. However, results were somewhat equivocal as to whether the same pattern was present for source memory errors once the discriminability of pair-mate faces was taken into account.

Across three experiments, we showed that higher levels of physical resemblance across episodes are associated with higher subsequent generalization across those episodes. This effect was present even when discarding the 75% blends that Experiment 3 indicated were not discriminable from one another. In fact, the 50% blends showed comparable levels of generalization with the 75% blends without the issue of poor discriminability. That is, participants generalized across the 50% blends at a rate similar to the condition where the participants could not even tell the pair-mate faces apart. Further, rates of generalization for 50% blends in Experiment 3 were comparable with memory for their respective trained associations, indicating that the participants generalized as well as could be expected given their memory for the premises. Together, these findings provide strong evidence that physical resemblance can help individuals make connections between related experiences, even when similar items are clearly discriminable from one another.

Prior studies of acquired equivalence have typically used stimuli without systematic resemblance between related experiences, akin to the no-shared and 1% conditions. Some of these studies have, nonetheless, found robust generalization (Edwards et al., 1982; Shohamy and Wagner, 2008; Meeter et al., 2009), while other studies found rates of generalization that were above chance but relatively low (Duncan et al., 2012; de Araujo Sanchez and Zeithamova, 2020). For instance, a study from our laboratory has previously shown in a large sample (*N* = 190) that rates of acquired equivalence can be quite modest: around 55% (when chance is 50%), with many subjects not showing any hint of generalization (de Araujo Sanchez and Zeithamova, 2020). Interestingly, we rarely saw above-chance rates of acquired equivalence at lower similarity levels (for the no-shared parent blends, 1% blends, or 25% blends), with the only exception being the 1% blend condition in Experiment 3. One possible explanation for lower generalization rates in conditions with low resemblance is that there may be generalization tradeoffs across quadruplets. If making connections between related experiences is cognitively demanding, then the participants may have been selective about when they made such links. In this way, physical resemblance may have served as a cue as to which experiences to link, promoting generalization for some pairs but inhibiting generalization for others. Future studies could manipulate pair-mate similarity between subjects to test whether the effect of physical resemblance is driven in whole or in part by the contrast between high and low resemblance within the same set.

Across the experiments, we found that generalization rates were consistently higher for the 50%/shared parent blends than the 1%/no shared parent blends. However, we did not find that rates of generalization for the 25% blends were intermediate between the 1 and 50% blends. Instead, generalization rates for the 25% blends were either comparable with the rate for the 1% blends (Experiment 2) or significantly lower than the 1% blends (Experiment 3). Matched generalization rates for 1 and 25% blends can be explained by the 25% blend level not generating a level of similarity that the participants could detect, like a mirror of the lack of discriminability we found between faces at the 75% blend level. However, the lower generalization rates for 25% blends compared with 1% blends were unexpected and more difficult to explain. Future studies will be needed to determine if this was simply due to sampling error or whether it is a real, replicable pattern, perhaps reflecting some form of pattern separation or repulsion effect (Chanales et al., 2017).

While we found that physical resemblance led to increases in generalization, research on reducing memory interference often shows that representations of highly similar items are orthogonalized to make them discriminable from one another (for reviews see Colgin et al., 2008; Yassa and Stark, 2011; Leal and Yassa, 2018). For example, Favila et al. (2016) showed that hippocampal representations for two highly similar scene images became more dissimilar from one another when paired with a common face, presumably to aid in discriminating between the scenes despite their shared perceptual details and shared association. The degree of integration vs. separation is driven to some extent by whether learning demands emphasize commonalities across related items or discrimination between them (Ashby et al., 2020; Chanales et al., 2020). During acquired equivalence learning in this study, instructions did not explicitly emphasize either discrimination between similar faces or generalization across them, and the participants were not aware of upcoming generalization or source memory tests. Under these conditions, it seems that the participants defaulted to linking across similar experiences in service of generalization.

While the data support the role of physical resemblance in promoting generalization, the support for the proposed mechanism—that similarity increases reactivation of related memories and leads to integration across the related experiences—is less clear. Prior studies of episodic inference, such as those using the acquired equivalence paradigm, have often focused on the role of memory integration in supporting generalization (Shohamy and Wagner, 2008; Zeithamova et al., 2012a; Schlichting et al., 2015). Such studies have shown that integrating representations of related episodes at the time of learning can facilitate later generalization, which tends to be faster and more accurate when based on integrated representations compared with separate representations (Shohamy and Wagner, 2008; Schlichting et al., 2014). Prior study has also identified several contextual factors that can increase integrative encoding of related episodes, such as explicit instructions to integrate (Richter et al., 2016), the temporal proximity of related episodes (Zeithamova and Preston, 2017), and whether related associations are studied in a blocked vs. intermixed manner (Schlichting et al., 2015). Here, we tested another such factor, physical resemblance, and found that rates of generalization were higher for faces with increased resemblance, consistent with the idea that similarity promotes integrative encoding, which in turn supports subsequent generalization. A similar effect of physical resemblance was recently reported in another inference paradigm, associative inference, where participants are explicitly asked to relate A and C items after separately learning A-B and B-C associations (Molitor et al., 2021). Thus, this aspect of the findings is consistent with the proposal that physical resemblance serves as a pattern completion cue to reactivate prior related episodes, which then becomes integrated with current experience.

However, not all aspects of the data point clearly to an integration mechanism. Prior study has shown that successful inference can lead to poorer source memory (Carpenter and Schacter, 2017, 2018). Anecdotal evidence from Shohamy and Wagner (2008) also suggested that successful generalization was accompanied by mistaking inferred face preferences for actually observed ones. These findings have been taken as evidence that integrating across episodes causes unique aspects of related experiences to be discarded, and assumptions that generalization and false memory may be two sides of the same coin (Zeithamova et al., 2012b; Varga et al., 2019). We thus expected to find increases in source memory errors with increasing rates of generalization, indicating that integration caused the loss of contextual information or that the participants mistakenly attributed internal reactivation of a pair-mate for an external presentation of the untrained association during encoding. When only considering blends where pair-mate faces were discriminable from one another, results for this aspect of the hypothesis were mixed. In Experiment 1, there was a significant increase in false source memories between the no shared parent and the shared parent pair-mates, and the increase mirrored the increase in generalization. This finding is in line with integration as the mechanism driving the effects of physical similarity. However, in Experiments 2 and 3, there was no effect of the blend level on source memory errors across the 1, 25, and 50% blends. Instead, rates of “both” responses were consistently lower for untrained associations compared with trained associations across blend levels. This contrasts with the generalization scores for 50% blends, which matched or nearly matched accuracy for trained associations, indicating that high generalization was not always accompanied by source memory confusion. Yet, complicating the story further, it is also not possible to conclude that the effect of physical similarity was different for generalization and source memory, as the difference across tests was not significant in any experiment. Instead, we are left with a clear effect of physical similarity in generalization and equivocal findings from source memory.

What might cause these mixed findings? Importantly, integrative encoding is but one mechanism proposed to support generalization. Other mechanisms of generalization have been postulated, with potentially distinct predictions about memory for individual events, memory generalization, and their relationship (Zeithamova and Bowman, 2020). Some argue that integrated representations are not needed; instead, generalization can be achieved on-demand, based on flexible retrieval of separate episodes (Kumaran, 2012; Kumaran and McClelland, 2012). The degree of physical similarity across pair-mates could potentially affect on-the-fly generalization from separate memories if the overlap across experiences increased the probability of successfully chaining all relevant memories. In this case, the generalization would not need to come at the expense of detailed memory for individual experiences. Indeed, our prior study that did not include a similarity manipulation showed that rates of generalization and source memory scores were not related either across subjects or on a trial-to-trial basis (de Araujo Sanchez and Zeithamova, 2020). Still, others have found that generalization can be positively related to the fidelity of individual memories (Banino et al., 2016). Thus, the chained retrieval of separate representations may have contributed to generalization in this study to some degree, explaining why high levels of generalization were not always accompanied by high levels of false memory.

Alternatively, similar predictions would stem from recent proposals that people may represent the same events at multiple levels of specificity, forming integrated representations alongside separate ones rather than at their expense (Collin et al., 2015; Schapiro et al., 2017; Brunec et al., 2018; Bowman et al., 2020; Zeithamova and Bowman, 2020). Different representations may be differentially susceptible to the effects of physical similarity, in which case the benefit of increased similarity for generalization (more likely relying on integrated memories) may not be accompanied by a corresponding effect on source memory (more likely relying on separate memories of individual events). While the current data are inconclusive with respect to whether generalization and source memory judgments were based on integrated memories, separated memories or both, they highlight the benefit of considering multiple measures in interpreting results rather than drawing conclusions about underlying mechanisms (such as integration) based on the generalization score alone. Importantly, the data clearly show that physical similarity promotes generalization across episodes, whether it is through promoting the formation of integrated representations and/or through enhancing flexible retrieval for on-the-fly generalization.

## Summary

In the three experiments, we manipulated the degree of overlap across related episodes in an acquired equivalence paradigm and tested the tendency to generalize across experiences and the ability to remember the source of generalized information. All three experiments showed increases in generalization for experiences with greater overlap but differed in whether errors in source memory accompanied increases in generalization. These results suggest a clear faciliatory effect of resemblance across episodes in the generalization that may sometimes, but not always, come with a loss of memory specificity.

## Data Availability Statement

The datasets presented in this study can be found in online repositories. The names of the repository/repositories and accession number(s) can be found at: https://osf.io/2ubgv/.

## Ethics Statement

The studies involving human participants were reviewed and approved by Research Compliance Services, University of Oregon. The patients/participants provided their written informed consent to participate in this study.

## Author Contributions

All the authors contributed to the conceptualization, design of the experiments, and edited the manuscript. M-AA, SR, and WH programmed the experiments and oversaw data collection. CB, M-AA, SR, and WH contributed to data analysis. CB wrote the first draft of the manuscript.

## Conflict of Interest

The authors declare that the research was conducted in the absence of any commercial or financial relationships that could be construed as a potential conflict of interest.

## Publisher's Note

All claims expressed in this article are solely those of the authors and do not necessarily represent those of their affiliated organizations, or those of the publisher, the editors and the reviewers. Any product that may be evaluated in this article, or claim that may be made by its manufacturer, is not guaranteed or endorsed by the publisher.
